# SARS‐CoV‐2 Vaccination is Not Associated With Involuntary Childlessness in Female Healthcare Workers: A Multicenter Cohort Study

**DOI:** 10.1111/irv.13333

**Published:** 2024-06-06

**Authors:** Tamara Dörr, Sabine Güsewell, Alexia Cusini, Angela Brucher, Stephan Goppel, Fabian Grässli, Elsbeth Betschon, J. Carsten Möller, Manuela Ortner, Markus Ruetti, Reto Stocker, Danielle Vuichard‐Gysin, Ulrike Besold, Lorenz Risch, Matthias von Kietzell, Matthias Schlegel, Stefan P. Kuster, Christian R. Kahlert, Philipp Kohler, for the SURPRISE (SURveillance of infectious diseases among health Professionals In SwitzErland) Study Group

**Affiliations:** ^1^ Division of Infectious Diseases and Hospital Epidemiology Cantonal Hospital St Gallen St Gallen Switzerland; ^2^ Division of Infectious Diseases Cantonal Hospital Graubünden Chur Switzerland; ^3^ Psychiatry Services of the Canton of St. Gallen (South) St Gallen Switzerland; ^4^ Ambulatory Services Psychiatry Services of the Canton of St. Gallen (North) St. Gallen Switzerland; ^5^ Department of Psychiatry Clienia Littenheid Littenheid Switzerland; ^6^ Center for Neurological Rehabilitation Zihlschlacht Switzerland; ^7^ Rheintal Werdenberg Sarganserland Hospital Group Grabs Switzerland; ^8^ Fuerstenland Toggenburg Hospital Group Wil Switzerland; ^9^ Hirslanden Clinic Zurich Switzerland; ^10^ Division of Infectious Diseases and Hospital Epidemiology Thurgau Hospital Group Muensterlingen Switzerland; ^11^ Department of Research and Development Swiss National Centre for Infection Prevention (Swissnoso) Berne Switzerland; ^12^ Geriatric Clinic St. Gallen St. Gallen Switzerland; ^13^ Labormedizinisches Zentrum Dr Risch Ostschweiz AG Buchs Switzerland; ^14^ Private Universität im Fürstentum Liechtenstein Triesen Liechtenstein; ^15^ Centre of Laboratory Medicine University Institute of Clinical Chemistry, University of Bern, Inselspital Bern Switzerland; ^16^ Hirslanden Clinic Stephanshorn St. Gallen Switzerland; ^17^ Department of Infectious Diseases and Hospital Epidemiology Children's Hospital of Eastern Switzerland St. Gallen Switzerland

**Keywords:** healthcare workers, involuntary childlessless, reproductive health, SARS‐CoV‐2, vaccination

## Abstract

**Background:**

There is debate about the causes of the recent birth rate decline in high‐income countries worldwide. During the pandemic, concern about the effects on reproductive health has caused vaccine hesitancy. We investigated the association of SARS‐CoV‐2 vaccination and infection with involuntary childlessness.

**Methods:**

Females in fertility age within a prospective multicenter cohort of healthcare workers (HCW) were followed since August 2020. Data on baseline health, SARS‐CoV‐2‐infection, and vaccination were obtained and regularly updated, in which serum samples were collected repetitively and screened for anti‐nucleocapsid and anti‐spike antibodies. In October 2023, participants indicated the presence of involuntary childlessness with onset during the pandemic, whereas those indicating an onset before the pandemic were excluded. The association of involuntary childlessness and SARS‐CoV‐2‐vaccination and infection was investigated using univariable and multivariable analysis. Sensitivity analysis was performed to compare those reporting involuntary childlessness with those birthing a child since 2020.

**Results:**

Of 798 participants, 26 (3.2%) reported involuntary childlessness starting since the pandemic. Of the involuntary childless women, 73.1% (19/26) were vaccinated compared to 86.0% (664/772) without involuntary childlessness (*p* = 0.73). SARS‐CoV‐2 infection was reported by 76.9% (20/26) compared to 72.4% (559/772) of controls (*p* = 0.64). Neither SARS‐CoV‐2 vaccination (aOR 0.91 per dose, 95%CI 0.67–1.26) nor infection (aOR per infection 1.05, 95%CI 0.62–1.71) was associated with involuntary childlessness. Sensitivity analysis confirmed these results.

**Conclusions:**

Among female HCW of fertility age, 3.2% indicated involuntary childlessness, which is comparable to pre‐pandemic data. No association between involuntary childlessness and SARS‐CoV‐2 vaccination or infection was found.

## 1 Introduction

With the introduction of the vaccine against severe acute respiratory syndrome coronavirus 2 (SARS‐CoV‐2) in early 2021, concerns about a possible negative impact on reproductive health have emerged. The novel mRNA‐transduction methods and similarities of vaccine components with placentar structures have been discussed to potentially impair fertility. Consecutively, fears about a negative impact on fertility have resulted in vaccine hesitancy especially in young women of reproductive age [1]. Also, the infection itself has been hypothesized to have an impact on structural elements of the reproductive system based on the expression of ACE2 receptors in the ovaries and endometrium [2].

The drop of birth rates since 2022 observed in many high‐income countries once again has given rise to concerns. In Switzerland, the birth rate increased slightly during 2021 but dropped by 8% in 2022 and another 4% in 2023, reaching a 20‐year low with 1.33 per woman [3, 4, 5]. The same trend has been noticeable in many other high‐income countries throughout the world [6, 7]. Germany, for instance, experienced a birth rate decline of 7.1% in 2022 [8], and similar figures have been observed in Canada [9] or the United States [10, 11, 12]. Due to the temporal context with the pandemic and especially the uptake of the SARS‐CoV‐2 vaccination in early 2021, some have proposed a causal relationship [13, 14]. As in many other European countries, Switzerland experienced the deadliest pandemic wave starting October 2020, its abatement in February 2021 having been attributed at least partly to the availability of the vaccination, when a large proportion of the Swiss population got vaccinated with the mRNA vaccines BNT162b2 and mRNA‐1273.

In light of these recent demographic developments and the concerns around SARS‐CoV‐2 vaccination and infection, we aimed to investigate the association of involuntary childlessness and SARS‐CoV‐2 vaccination in women of fertility age in our cohort of healthcare workers (HCW).

## 2 Materials and Methods

We performed a nested cross‐sectional study in our prospective multicenter cohort, which includes HCW with and without patient contact from northern and eastern Switzerland, as previously described [15]. In short, HCW were recruited in nine healthcare networks since August 2020 and prospectively followed. Baseline data (i.e., anthropometrics, determinants of health, occupation, and social life) were obtained by means of an online questionnaire at date of inclusion, self‐reported data on laboratory‐confirmed SARS‐CoV‐2 infections (i.e., positive polymerase chain reaction test and/or positive antigen test) and SARS‐CoV‐2 vaccinations (i.e., number, date, type) were collected in regular follow‐up questionnaires.

In October 2023, participants answered questions regarding childlessness (i.e., presence, onset, involuntary vs. voluntary, evolution of childbearing preferences during the pandemic, reasons for a change if present). As described earlier, participants additionally provided serum samples that were screened for anti‐nucleocapsid (anti‐N) antibodies and anti‐spike (anti‐S) antibodies [15]. The study was approved by the Ethics Committee of Eastern Switzerland.

For this analysis, we only included participants identifying as females in fertility age, which we defined as 16–45 years. Our main outcome was involuntary childlessness with onset during the pandemic, defined as 2020 or later. Women reporting no date of onset were included into this group, whereas those indicated onset before the pandemic were excluded. To assess the extent of a potential selection bias, we compared baseline characteristics, SARS‐CoV‐2 infection, and vaccination status as of October 2023, as well as anti‐N and anti‐S titers between those who opted to answer the questions on involuntary childlessness and those that did not. For our main analysis, we compared the same parameters between those with and without involuntary childlessness. The questions on childbearing intentions were analyzed descriptively. For statistical analysis, we used univariable and multivariable logistic regression analysis. For the multivariable model, we selected the following variables a priori: age, number of SARS‐CoV‐2 vaccinations, and number of positive SARS‐CoV‐2 swabs reported since start of the pandemic until October 2023 (with two different swabs being at least 1 month apart). A sensitivity analysis using vaccination and infection as binary variables, respectively, was performed. We also performed a sensitivity analysis with the same group of women with involuntary childlessness, but with those who reported birthing a child since 2020 as control group. Odds ratios (OR) and corresponding 95% confidence intervals (95% CI) were calculated. Fisher's exact test was used for calculating *p* values of main outcomes, presuming significance at *p* < 0.05. All analyses were performed with R statistical software Version 4.2.2, and EQUATOR reporting guidelines including STROBE for observational studies were adhered to.

## 3 Results

Of 1824 female participants in our cohort, we identified 886 female participants in fertility age, 803 (90.6%) of which answered the questions on involuntary childlessness. Characteristics between those answering and those not answering the questionnaire were similar (Table S1). However, compared to women not answering the questionnaire (*n* = 83), respondents were more likely to ever report a positive SARS‐CoV‐2 result (*p* < 0.05) and had lower median anti‐N‐titers in October 2023 (*p* < 0.01).

Of 803 women answering the questionnaire, 26 (3.2%) reported involuntary childlessness with an onset since the pandemic. Another five reported longer‐standing of their unfulfilled desire for a child and were thus excluded from further analysis. Of those with onset since the pandemic, 73.1% (19/26) were vaccinated against SARS‐CoV‐2 compared to 86.0% (664/772) of controls (*p* = 0.73), with no difference in mean number of vaccinations between groups. Almost all individuals were vaccinated with an mRNA vaccine (657/683, 96.2%). Among those with involuntary childlessness, 76.9% (20/26) reported at least one SARS‐CoV‐2 infection compared to 72.4% (559/772) of the control group (*p* = 0.64), with again no difference in mean number of infections. Although age and the presence of a comorbidity or of post‐acute COVID‐19 sequelae were similarly distributed between groups, involuntary childless women were more likely to work as physician (15.4% vs. 9.3%) or in administration (23.1% vs. 13.2%) but less likely to work as nurse (56.1% vs. 42.3%) compared to those without involuntary childlessness (Table 1).

**TABLE 1:** Baseline characteristics of women reporting on involuntary childlessness (*n* = 798).

	Women reporting no involuntary childlessness	Involuntarily childless women since 2020	*p* value^a^
*n*	772	26	
Age (in years), median (IQR)	36 (31–41)	35 (32‐39)	0.77
≥ 1 comorbidity (%)	375 (48.6)	13 (50.0)	1.00
Profession			0.41
Physician (%)	72 (9.3)	4 (15.4)	
Nurse (%)	433 (56.1)	11 (42.3)	
Therapist (%)	46 (6.0)	2 (7.7)	
Administrative worker (%)	102 (13.2)	6 (23.1)	
Other (%)	119 (15.4)	3 (11.5)	
SARS‐CoV‐2 vaccination			0.73
Unvaccinated	108 (14.0)	7 (26.9)	
1 or 2 vaccinations (%)	214 (27.7)	3 (11.5)	
3 vaccinations (%)	351 (45.5)	13 (50.0)	
≥ 4 vaccinations (%)	99 (12.8)	3 (11.5)	
SARS‐CoV‐2 infections			0.64
No positive swab (%)	213 (27.6)	6 (23.1)	
1 positive swab (%)	395 (51.2)	14 (53.8)	
≥ 2 positive swabs (%)	164 (21.2)	6 (23.1)	
Anti‐N titer, median (IQR)	53 (6.4–173)	167 (23.35–214)	0.18
Anti‐S titer, median (IQR)	5000 (3838.2–5000)	4774 (1110.5–5000)	0.14
PASC (%)	46 (6.2)	2 (8.0)	1.00

Abbreviations: Anti‐N = anti‐nucleoside antibodies, Anti‐S = anti‐spike antibodies, IQR = interquartile range, PASC = post‐acute sequelae of COVID‐19, self‐perception, SARS‐CoV‐2 = severe acute respiratory syndrome coronavirus type 2.

^**a**^ Calculation for categorical variables using chi^2^‐test; calculation for continuous variables using Kruskal–Wallis test.

**FIGURE 1:**
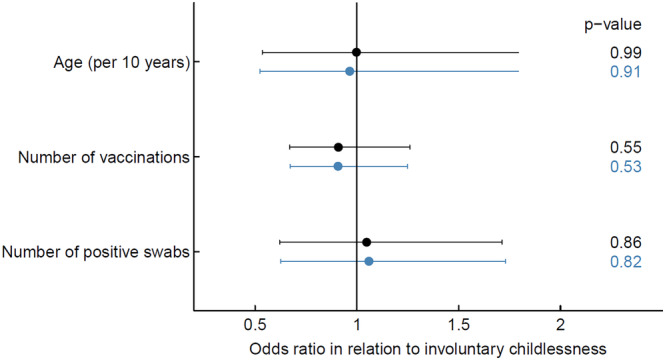
Association of involuntary childlessness with age, number of vaccinations, and number of positive SARS‐CoV‐2 swabs. Results of univariable analysis in blue; results of multivariable logistic regression analysis in black.

SARS‐CoV‐2 vaccination was not associated with involuntary childlessness, neither in univariable (OR 0.91 per vaccine dose, 95% CI 0.67–1.25) nor in multivariable analysis (adjusted OR 0.91 per vaccine dose, 95% CI 0.67–1.26). Also, no association could be observed for previous infection (adjusted OR per infection 1.05, 95% CI 0.62–1.71) (Figure 1). This was also true when SARS‐CoV‐2 vaccination and infection were used as binary variables with an aOR of 1.36 (95% CI 0.57–3.79) for infection and 0.42 (95% 0.18–1.12) for vaccination, respectively. Sensitivity analysis with women who gave birth to a child during the pandemic as controls showed similar results (Table S2 and Figure S1).

**FIGURE 2:**
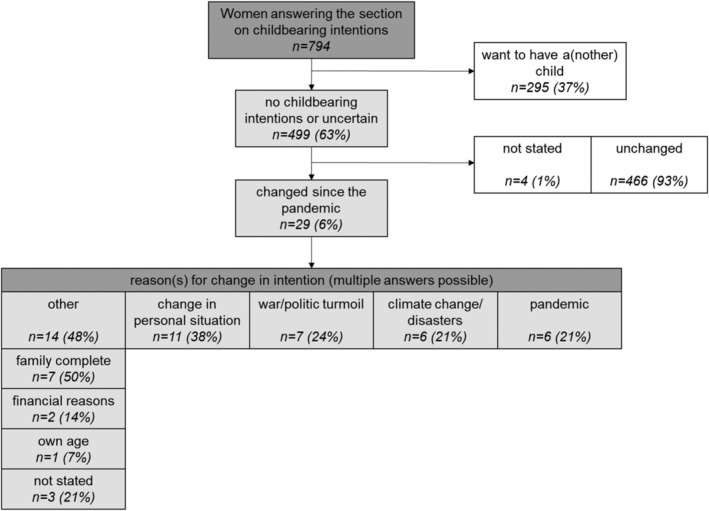
Childbearing intentions and their evolution since the pandemic.

The questions on childbearing preferences and their evolution during the pandemic were answered by 794 women. Of these, the majority (499/794, 63%) did report no childbearing intentions. Thereof, 6% (29/499) reported that they lost their desire to bear a child since the pandemic. As reasons, a change in personal situation (38%) or other reasons (48%) were stated most commonly, 50% of the latter being specified as completion of family planning during the pandemic. Other reasons stated were the pandemic itself (6/29), environmental disasters (6/29), and current political turmoils (7/29) (Figure 2).

## 4 Discussion

In light of dropping birth rates in Western countries, we investigated involuntary childlessness among female participants of a Swiss HCW cohort and its association with SARS‐CoV‐2 vaccination and infection. Our results indicate no association of these predictors with involuntary childlessness. The most important reasons for change in childbearing preference during the pandemic were—besides personal circumstances and the completion of family planning—the pandemic itself, climate change, and political turmoils.

In our cohort, 3% of female HCW in fertility age indicated involuntary childlessness since the start of the pandemic. Based on the estimation that only 10% of those affected seek treatment, this is comparable to pre‐pandemic data in the Swiss population from 2015 to 2019, where 0.25%–0.38% of females in this age range sought fertility treatment [16, 17]. An older study estimated the lifetime prevalence of infertility at 2.8% [18] based on a cross‐sectional survey of women at age 29 or older.

In the present investigation, we could not find an association of involuntary childlessness with the SARS‐CoV‐2 vaccination, which is in line with the findings of fertility research. In women undergoing fertility treatment, no differences in follicular fluid composition, follicle quality, hormonal profiles, or indicators of ovarian reserve could be found in those vaccinated compared to those unvaccinated [19, 20, 21, 22]. These as well as our results support the safety of the SARS‐CoV‐2 vaccine regarding female reproductive health. The same has been reported for their male counterparts, where no association of male infertility with SARS‐CoV‐2 vaccination could be found [23, 24].

Also, regarding SARS‐CoV‐2 infections, we did not see an association with involuntary childlessness. Although shared immune determinants of SARS‐CoV‐2 with proteins involved in oocyte development led to concerns about a potential detrimental effect on female reproductive health [25], observational studies could not show infection‐related differences in quantity or quality of oocytes [22, 26, 27]. Several studies found an association of SARS‐CoV‐2 infection with alterations of menstrual cycle length and bleeding intensity [28], which has been attributed to the function of angiotensin II and ACE 2 in both the infection and endometrium physiology. However, these changes were temporary, and no long‐term effects were observed [28, 29, 30]. Similarly, SARS‐CoV‐2 infection in males has been shown to potentially impair semen quantity, quality, and functional parameters. However, analogically to females' reproductive health parameters, these changes are temporary, and no long‐term effects could be demonstrated [31, 32].

Therefore, other factors in connection to the pandemic must be taken into account when exploring the association of birth rate decline and the pandemic. Especially during the initial phases of the pandemic, there was substantial variation in birth rate development when comparing high‐income countries [33], which has been associated with the onset of containment measures [7]. Historically, such containments have resulted in a birth rate incline [7], which could also be documented for the first pandemic year in some countries including Switzerland [33, 34]. However, as the overall trend shows a substantial decline, the crisis itself and its socioeconomic impact seem to play a role. In fact, an impact on birth rates has been observed in association with general crises in the past, not only of health‐related but also of economic and environmental nature [35]. For the SARS‐CoV‐2 pandemic, De Geyter et al. [7] found the birth rate decline to be most pronounced in those countries with the highest excess mortality, which supports feelings of insecurity as a mechanism, as proposed earlier [36]. Although the small number of participants reporting a change of childbearing preference demands cautionary interpretation, this is also reflected in our exploratory data where economic and political insecurities as well as the pandemic itself have been stated as reasons for the decision against future children. Likewise, Adelman et al. [37] have shown the variation of birth rates in US states to be influenced stronger by economic, racial, political, and social factors than the severity of the first or second wave of the SARS‐CoV‐2 pandemic.

### 4.1 Strengths and Limitations

The population of our study is well defined, providing a robust data source for the present investigation. Additionally, the validation of vaccination status that had been conducted earlier [38] as well as including serological testing strengthens the external validity of our results by verifying the self‐reported data. However, our study has limitations. First, these data are from a HCW cohort. Other investigators have shown the workplace to have a significant impact on female sexual health with the most impact on those who were not able to work outside their home [39]. As for most HCW, their workplace did not change; this may limit the generalizability to the general population, although it is not clear how the potential influence on sexual function may translate into involuntary childlessness. Additionally, health literacy and awareness may be different in HCW compared to the general population and could introduce confounding. Second, the sample size of involuntary childless women is small, as is the proportion of women reporting a change in childbearing preferences. Therefore, only few variables could be accounted for in multivariable analysis, and the analysis of potential drivers of preference changes is only exploratory. Third, involuntary childless attributable to male fertility problems is not taken into account. However, male infertility would also translate into women reporting involuntary childlessness, which would have been captured in our investigation. These limitations should be kept in mind when interpreting our results.

## 5 Conclusions

In our cohort of female Swiss HCW in fertility age, we documented a prevalence of involuntary childlessness of 3.2%, which is comparable to pre‐pandemic data. We found no association with SARS‐CoV‐2 vaccination or infection, which suggests other factors contributing to the decline of birth rates in high‐income countries. This adds to the evidence of the SARS‐CoV‐2 vaccination being safe regarding reproductive health.

## Author Contributions

**Tamara Dörr:** conceptualization, writing–original draft, visualization, formal analysis. **Sabine Güsewell:** conceptualization, writing–review and editing, visualization, validation, methodology. **Alexia Cusini:** investigation, writing–review and editing. **Angela Brucher:** investigation, writing–review and editing. **Stephan Goppel:** investigation, writing–review and editing. **Fabian Grässli:** data curation, formal analysis. **Elsbeth Betschon:** investigation, writing–review and editing. **J. Carsten Möller:** investigation, writing–review and editing. **Manuela Ortner:** investigation, writing–review and editing. **Markus Ruetti:** investigation, writing–review and editing. **Reto Stocker:** investigation, writing–review and editing. **Danielle Vuichard‐Gysin:** investigation, writing–review and editing. **Ulrike Besold:** investigation, writing–review and editing. **Lorenz Risch:** investigation, writing–review and editing. **Matthias von Kietzell:** investigation, writing–review and editing. **Matthias Schlegel:** investigation, conceptualization, writing–review and editing. **Stefan P. Kuster:** writing–review and editing, project administration, supervision; methodology. **Christian R. Kahlert:** writing–review and editing, conceptualization, project administration. **Philipp Kohler:** supervision, project administration, formal analysis, visualization, validation, writing–review and editing, funding acquisition, investigation, conceptualization. **(SURveillance of infectious diseases among health Professionals In SwitzErland) Study Group SURPRISE:** investigation, resources.

## Ethics Statement

The study was approved by the Ethics Committee of Eastern Switzerland.

## Conflicts of Interest

The authors declare no conflicts of interest.

### Peer Review

The peer review history for this article is available at https://www.webofscience.com/api/gateway/wos/peer‐review/10.1111/irv.13333.

## Supporting information


**Figure S1.** Association of involuntary childlessness with age, number of vaccinations and number of positive SARS‐CoV‐2 swabs in sensitivity analysis compared to women having birthed a child since 2020. Results of univariable analysis in blue, results of multivariable logistic regression analysis in grey.
**Table S1.** Baseline characteristics of women answering the questionnaire on childbearing preferences (*n* = 886).
**Table S2.** Baseline characteristics of women birthing a child since 2020 (*n* = 122).

## Data Availability

The datasets used in this study are not publicly available as they contain health‐related data. Individual participant data and statistical analysis plan will be available from the corresponding author upon reasonable request.
