# Supplementary material for: SARS‐CoV‐2 Vaccination is Not Associated With Involuntary Childlessness in Female Healthcare Workers: A Multicenter Cohort Study
Source: Influenza Other Respir Viruses. 2024 Jun 6;18(6):e13333. doi: 10.1111/irv.13333 (PMC11157148; doi:10.1111/irv.13333)
Supplement: Supplementary file 1 — Figure S1. Association of involuntary childlessness with age, number of vaccinations and number of positive SARS‐CoV‐2 swabs in sensitivity analysis compared to women having birthed a child since 2020. Results of univariable analysis in blue, results of multivariable logistic regression analysis in grey. Table S1. Baseline characteristics of women answering the questionnaire on childbearing preferences (n = 886). Table S2. Baseline characteristics of women birthing a child since 2020 (n = 122). [file IRV-18-e13333-s001.docx]

**Supplementary material**


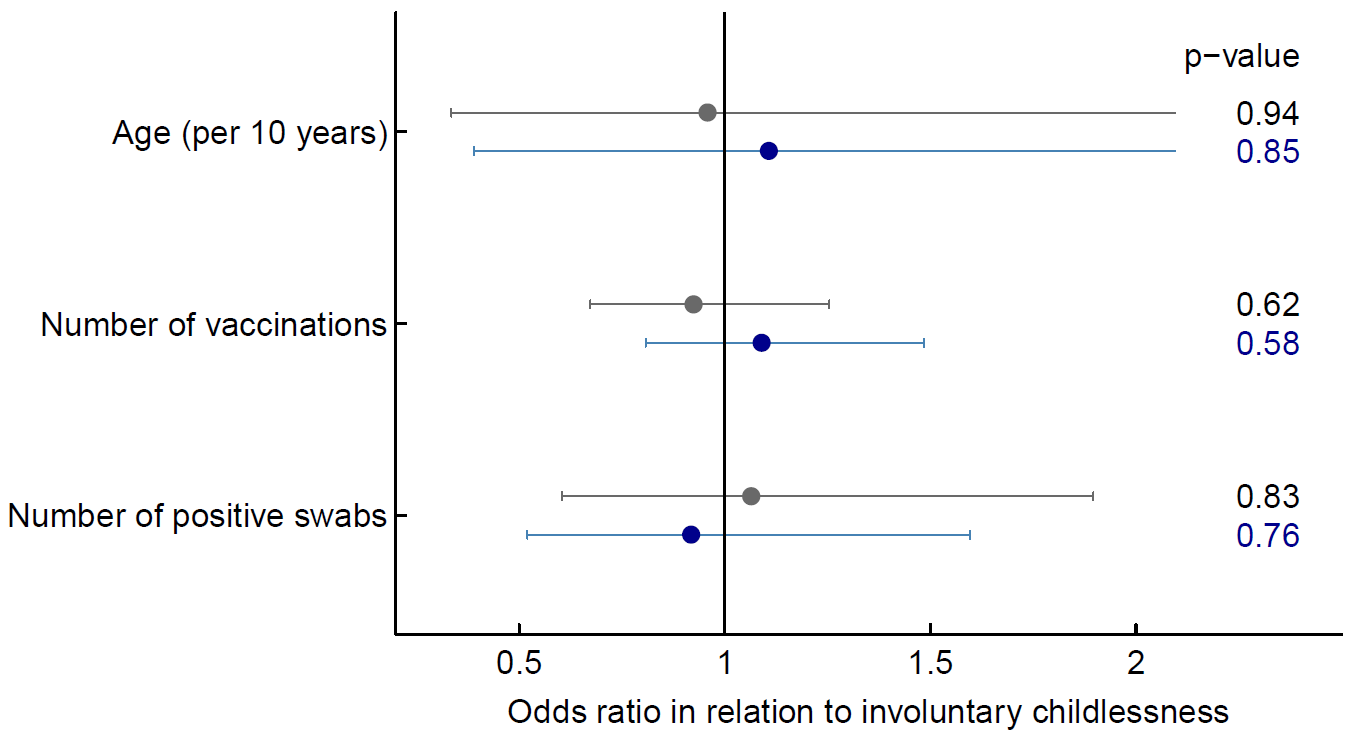


**Figure S1.** Association of involuntary childlessness with age, number of vaccinations and number of positive SARS-CoV-2 swabs in sensitivity analysis compared to women having birthed a child since 2020. Results of univariable analysis in blue, results of multivariable logistic regression analysis in grey.

**Table S1.** Baseline characteristics of women answering the questionnaire on childbearing preferences (n=886).

|  | Women answering the questions on childbearing preferences | | Women not answering the questions on childbearing preferences | | *p-value* ^‡^ |
| --- | --- | --- | --- | --- | --- |
| n | 803 **^†^** | | 83 | |  |
| Age (in years), median (IQR) | 36 | (31-41) | 32 | (30-39.5) | *0.06* |
| ≥ 1 Comorbidity (%) | 390 | (48.6) | 41 | (49.4) | *0.98* |
| Profession |  |  |  |  | *0.66* |
| Physician (%) | 76 | (9.5) | 7 | (8.4) |  |
| Nurse (%) | 447 | (55.7) | 50 | (60.2) |  |
| Therapist (%) | 49 | (6.1) | 3 | (3.6) |  |
| Administrative worker (%) | 109 | (13.6) | 8 | (9.6) |  |
| Other (%) | 122 | (15.2) | 15 | (18.1) |  |
| SARS-CoV-2 vaccination |  |  |  |  | *0.47* |
| unvaccinated | 117 | (14.6) | 11 | (13.3) |  |
| 1 or 2 vaccinations (%) | 217 | (27.0) | 24 | (28.9) |  |
| 3 vaccinations (%) | 366 | (45.6) | 44 | (53.0) |  |
| ≥ 4 vaccinations (%) | 103 | (12.8) | 4 | (4.8) |  |
| SARS-CoV-2 infections |  |  |  |  | ***<0.05*** |
| no positive swab (%) | 221 | (27.5) | 13 | (15.7) |  |
| 1 positive swab (%) | 411 | (51.2) | 49 | (59.0) |  |
| ≥ 2 positive swabs (%) | 171 | (21.3) | 21 | (25.3) |  |
| Anti-N-Titer, median (IQR) | 57 | (6.4-178) | 141 | (75-208) | ***<0.01*** |
| Anti-S-Titer, median (IQR) | 5000 | (3753.2-5000) | 5000 | (4486-5000) | *0.85* |
| PASC (%) | 48 | (6.3) | 2 | (12.5) | *0.63* |

IQR = interquartile range

SARS-CoV-2 = severe acute respiratory syndrome coronavirus type 2

Anti-N = anti-nucleosid-antibodies

Anti-S = anti-spike-antibodies

PASC = post-acute sequelae of COVID-19, self-perception

**^†^** five of these indicated involuntary childlessness with onset before the pandemic and were excluded from further analyses

^‡^ calculation for categorical variables using chi^2^-test, calculation for continuous variables using kruskal-wallis-test

**Table S2. Baseline characteristics of women birthing a child since 2020 (n=122)**

|  | Women birthing a child since 2020 | | Involuntarily childless women since 2020 | | | *p-value* ^†^ | |
| --- | --- | --- | --- | --- | --- | --- | --- |
| n | 96 | | 26 | | |  | |
| Age (in years), median (IQR) | 35 | (32.8-38) | 35 | (32-39) | | *0.95* | |
| ≥ 1 Comorbidity (%) | 44 | (45.8) | 13 | (50.0) | | *0.88* | |
| Profession |  |  |  |  | | *0.20* | |
| Physician (%) | 13 | (13.5) | 4 | (15.4) | |  | |
| Nurse (%) | 54 | (56.2) | 11 | (42.3) | |  | |
| Therapist (%) | 6 | (6.2) | 2 | (7.7) | |  | |
| Administrative worker (%) | 7 | (7.3) | 6 | (23.1) | |  | |
| Other (%) | 16 | (16.7) | 3 | (11.5) | |  | |
| SARS-CoV-2 vaccination |  |  |  |  | | *0.58* | |
| unvaccinated | 22 | (22.9) | 7 | | (26.9) |  | |
| 1 or 2 vaccinations (%) | 31 | (32.3) | 3 | | (11.5) |  | |
| 3 vaccinations (%) | 29 | (30.2) | 13 | | (50.0) |  | |
| ≥ 4 vaccinations (%) | 14 | (14.6) | 3 | | (11.5) |  | |
| SARS-CoV-2 infections |  |  |  |  | | *0.89* | |
| no positive swab (%) | 26 | (27.1) | 6 | | (23.1) |  | |
| 1 positive swab (%) | 42 | (43.7) | 14 | | (53.8) |  | |
| ≥ 2 positive swabs (%) | 28 | (29.2) | 6 | | (23.1) |  | |
| Anti-N-Titer, median (IQR) | 64 | (6.4-198) | 167 | (23.35-214) | | | *0.39* |
| Anti-S-Titer, median (IQR) | 5000 | (2609-5000) | 4774 | (1110.5-5000) | | *0.47* | |
| PASC (%) | 2 | (2.1) | 2 | (8) | | *0.41* | |

IQR = interquartile range

SARS-CoV-2 = severe acute respiratory syndrome coronavirus type 2

Anti-N = anti-nucleosid-antibodies

Anti-S = anti-spike-antibodies

PASC = post-acute sequelae of COVID-19, self-perception

**^†^** calculation for categorical variables using chi^2^-test, calculation for continuous variables using kruskal-wallis test
